# Circulating expression of Hsa_circRNA_102893 contributes to early gestational diabetes mellitus detection

**DOI:** 10.1038/s41598-020-76013-5

**Published:** 2020-11-04

**Authors:** Hongling Yang, Weitao Ye, Ruihua Chen, Fangling Zeng, Yan Long, Xiaoyan Zhang, Jun Ma, Qiangsheng Gan, Rehemayi Rehemutula, Chunyan Zhu

**Affiliations:** 1 Department of Laboratory, Guangzhou Women and Children’s Medical Centre, Guangzhou Medical University, Guangzhou, People’s Republic of China; 2 Department of Preventive Medicine, School of Public Health, Guangzhou Medical University, Xinzao, Panyu District, Guangzhou, 511436 People’s Republic of China; 3 Department of Health Education, Health Education Center of Panyu, Guangzhou, People’s Republic of China

**Keywords:** Gestational diabetes, Biomarkers

## Abstract

Due to a poor availability of reliable biomarkers, detecting gestational diabetes mellitus (GDM) in early pregnancy remains a challenge. Novel biomarkers like Circular RNAs (circRNAs) may be a promising diagnostic tool. The aim of this study was (a) to identify circRNAs deregulated in GDM and (b) evaluate the potential of circRNAs in detecting GDM. The circRNAs expression profiling in 6 paired women (with and without GDM) was measured by microarray. The levels of five most relevant circRNAs were validated in 12 paired participants by qRT-PCR. To verify the reproducibility of qRT-PCR, significantly differential expressed circRNA levels were confirmed in 18 paired participants. A receiver operating characteristic (ROC) curve was used to evaluate the diagnostic value. The areas under ROC curves of hsa_circRNA_102893 were 0.806 (95% CI 0.594–0.937) and 0.741 (0.568–0.872) in training set and test set, respectively. Circulating circRNAs reflect the presence of GDM. Hsa_circRNA_102893 may be a potential novel and stable noninvasive biomarker for detecting GDM in early pregnancy.

## Introduction

Gestational diabetes mellitus (GDM) is a common metabolic complication of pregnancy, and is a leading cause of perinatal complications^1,2^. Despite the extensive research investigating the pathogenesis of GDM, the primary causes of this disease are unknown. While there are well-recognized risk factors for GDM such as advanced maternal ages, family history of diabetes and obesity^3^, the identification of common genetic variants associated with GDM has proven difficult despite strong evidence that susceptibility is heritable.

To explore the molecular mechanism of GDM, many studies have focused on the differences in gene expression between pregnant women with GDM and pregnant women with normal glucose tolerance (NGT)^4–6^. The results of these studies indicate that changes in placental gene expression are involved in the occurrence of GDM. However, the regulatory mechanisms that control these genetic changes remain largely unknown.

The biological functions of circRNAs remain to be elucidated. Until very recent years, circular RNAs (circRNAs) have emerged as important regulators of gene expression in some disease related pathologies. Circular RNAs (circRNAs) are a special class of endogenous RNA molecules with covalently joined 3′- and 5′-ends formed by back-splice events thus presenting as covalently closed continuous loops^7^. The important features of circRNAs include high abundance, great diversity, stable structure, and high tissue-specific expression^7,8^. The function of animal circRNAs is speculated to serve as antagonists of specific miRNAs by functioning as miRNA sponges, such as circRNAs CDR1as (ciRS-7) and SRY^9,11^. Recent evidences demonstrate that circRNAs are involved in the development of several diseases, such as atherosclerosis^10^, cancer^11–13^, Diabetes mellitus^14^ and nervous system disorders^15,16^. However, little is known about circRNAs expression of GDM, which would show if there are new biomarkers serving as potential diagnosis and treatment targets for GDM.

In the current study, we first identified circRNAs deregulated in GDM and analyzed their potential as noninvasive biomarkers for early detection of GDM.

## Materials and methods

### Study participants

The participants were recruited from the Pregnancy Metabolic Disease and Adverse Pregnancy Outcome (PMDAPO) study^17^, which is an ongoing large prospective observational cohort study of pregnant women in Guangzhou, China. The pregnant women were recruited into the study at their first antenatal visit by a clinical research nurse at Guangzhou Women and Children’s Medical Centre. All the participants were followed up from the first antenatal care visit until delivery. From April 2015 to July 2016, a total of 842 pregnant women were recruited to the study during their first antenatal visit by a clinical research nurse. All women enrolled in this study were singleton pregnancy and delivered a live birth. Subjects with pre-existing diabetes, a hypertensive disorder complicating their pregnancy, pre-eclampsia or a history of multiple pregnancies were excluded from the study. The protocol for this study was approved by the ethics review committees of Guangzhou Medical University, and all study participants provided their voluntary signed informed consent. All procedures performed in this study involving human participants were in accordance with the ethical standards of the ethics review committees of Guangzhou Medical University and with the Helsinki declaration.

### Study design

All the participants were from the same cohort and followed up starting from their first antenatal care visit until delivery (Table 1). GDM was diagnosed after overnight fasting followed by a 75 g glucose load at—24 and 28 weeks gestation, according to the American Diabetes Association diagnostic criteria published in the pregnancy guidelines in the Standards of Medical Care in Diabetes, 2011^18^. Pregnant women were diagnosed with GDM if one or more of the following glucose levels were elevated: fasting ≥ 5.1 mmol/L, 1 h ≥ 10.0 mmol/L, and 2 h ≥ 8.5 mmol/L^18^.

**Table 1:** Summary of the participants in this study.

Phase	Group	Gestational weeks (week)	n	Platform
Cohort study	Pregnant women with GDM	8–41	106	–
	Healthy pregnant women	8–41	630	
Discovery	Pregnant women with GDM	15–24	6	Microarray
	Healthy pregnant women	15–24	6	
Training	GDM	15–24	12	qRT-PCR
	Control	15–24	12	
Test	GDM	15–24	18	qRT-PCR
	Control	15–24	18	

At first, a prospective cohort study was carried out to identify the risk factors of sociodemographic characteristics, clinical variables and biological chemistry parameters of GDM. Among the 736 subjects recruited, 106 were diagnosed with GDM after overnight fasting followed by a 75 g glucose load at ~ 24 and 28 weeks gestation.

Then, according to a nest case–control design, an equal number of matched, normal (non-GDM) control subjects was randomly selected from the group of pregnant women. The normal control subjects were matched with GDM women for maternal age, gestational weeks, gravidity and parity. According to the time sequence of registration, the control subjects were selected at random by the same nurse after delivery to ensure the controls did not have GDM, hypertensive disorders or pre-eclampsia during the gestation period.

To obtain reliable results, we conducted three sets (discovery set, training set and test set) of experiments, including a microarray test and twice validations by qRT-PCR. To identify the circRNAs microarray expression profiling of GDM in human peripheral blood, we explored 6 paired of samples (discovery set, 6 pregnant women with GDM and 6 healthy pregnant women) with microarray. Then, according to the results of discovery set, levels of five most relevant circRNAs were selected to validated in 12 paired particapnts (traning set) by qRT-PCR, Finally, according to the results of training set, the significant circRNAs were confirmed again in a test set (18 paired paricipants) of specimens by qRT-PCR to verify the reproducibility.

### Data collection

At their first antenatal visit, all pregnant women were asked to complete a questionnaire on maternal age, gravidity, parity, cigarette smoking during pregnancy, and family history of diabetes. The height and weight of each subject measured while wearing light clothing and no shoes were used to calculate body mass index. Weight was measured to the nearest 0.1 kg using a digital electronic scale, and height was measured to the nearest 0.1 cm using a stadiometer. Body mass index (BMI) was determined at the first antenatal visit, and calculated as weight divided by height squared (kg/m^2^). The maternal outcomes, fetal and neonatal information on prenatal problems and delivery were collected from the medical records.

### Blood collection

Peripheral venous blood samples (3 ml) of these participants in three sets (discovery set, training set and test set) were all collected with EDTA tubes during 15–24 weeks gestation after a 12 h overnight fast. These samples were immediately centrifuged at 3000 r/min at room temperature for 10 min within 1 h of collection. Then, the plasma was obtained and immediately stored at – 80 °C.

### Total RNA preparation

Total RNA was isolated from 250 mL plasma with TRIzol LS Reagent (Invitrogen, Karlsruhe, Germany) according to the manufacturer’s instructions. The quality of RNA was assessed by OD260 with NanoDrop ND-1000 Spectrophotometer (NanoDrop, Wilmington, DE, USA). The integrity of RNA was assessed by electrophoresis on a denaturing agarose gel.

### RNA labeling and CircRNA hybridization

Sample labeling and array hybridization were performed according to the manufacturer’s instructions (Arraystar Inc.). Briefly, circRNA were treated with Rnase R (Epicentre, Inc.) to remove linear RNAs. Then, each sample was amplified and transcribed into fluorescent labeled cRNA utilizing a random priming method with a Super RNA Labeling Kit (Arraystar, Rockville, Maryland, USA). The labeled cRNAs were purified by RNeasy Mini Kit (Qiagen).The concentration and specific activities of the labeled cRNAs (pmol Cy3/μg cRNA) were measured by NanoDrop ND-1000. CircRNA microarray hybridization was performed according to the manufacturer's protocol of Arraystar Human CircRNA Array (6 × 7 K, Arraystar, Rockville, Maryland, USA).

### CircRNA microarray data analysis

Scanned images were imported into Pro Gene Pix 6 (Axon) software for raw data extraction. Quantile normalization of raw data and subsequent circRNA expression profile analyze were performed using the R software package (R version 3.1.2).

CircRNAs differentially expressed between women with GDM and non-GDM, were conveniently estimated by fold-change filtering and t test. CircRNAs exhibiting fold changes ≥ 2.0 and p-values ≤ 0.05 were selected as significantly differentially expressed circRNAs. Hierarchical Clustering was performed to show the distinguishable circRNAs expression pattern among groups. The circRNA/microRNA interaction was predicted with Arraystar's home-made miRNA target prediction software. The differentially expressed circRNAs were annotated in detail with the circRNA/miRNA interaction information. The differentially expressed circRNAs were imported into the Gene Onotology database and KEGG PATHWAY database, respectively, and mapped into database genes. The functions involved in these circRNA sets were annotated and enrichment analysis of the pathways involved in these gene sets was conducted.

### Validation of candidate circRNAs using qPCR

Total RNA was extracted from specimens using TRIzol reagent (Invitrogen Life Technologies Corporation, Karlsruhe, Germany), and then the quantitative real-time reverse transcription-polymerase chain reaction (qPCR) was achieved to quantified the expression of the most relevant circRNAs using the the GoTaq qPCR Master Mix (Promega) on a ViiA 7 Real-time PCR System (Applied Biosystems) following the manufacturer's instructions. qPCR was applied to plasma samples from the validation set to validate the most relevant circRNAs.

Five significantly expressed circRNAs (hsa_circRNA_100380, hsa_circRNA_101232, hsa_circRNA_102736, hsa_circRNA_102893 and hsa_circRNA_103740) were evaluated in 12 paired participants (training set). The primers used in this study were shown in (Table S1). Significantly differential expressed circRNA levels in validation set were confirmed in a test set.

All samples were done in triplicate, and the result normalization across samples was performed using a median normalization procedure in each triplicate. The target gene and the housekeeping gene of each sample were subjected to a Real-time PCR reaction, respectively. According to the standard curve of gradient dilution DNA, the concentration of the target gene and housekeeping genes of each sample were directly generated by the machine. The concentration of the target gene for each sample is divided by the concentration of the housekeeping gene, which is the corrected relative amount of this gene for this sample.

### GDM-associated marker analysis

To identify if the candidate circRNAs validated by qPCR could better discriminated GDM from healthy pregnant women in different gestational weeks, we evaluated the performance of these circRNAs using the samples from the training set and test set, respectively. Diagnostic sensitivity and specificity values, and ROC curves and AUCs with corresponding 95% CIs were estimated. GDM-associated circRNA-targeted miRNA-mRNA network were predicted and annotated using Targetscan and miRanda.

### Statistical analysis

Statistical measures and tests were applied according to data distributions using SPSS software 16.0 (SPSS Inc., Chicago, IL, USA). All tests were two-tailed and p < 0.05 was considered statistically significant. The continuous variables were presented as the mean ± SD and the categorical variables were as frequency (%).

Bivariate logistic regression analyses were used to determine the influence of sociodemographic characteristics, clinical variables and biological chemistry parameters on GDM outcomes. In the cohort study, participants’ characteristics were firstly compared between the groups by two-tailed chi-square or t tests, and then a multivariable logistic regression analysis was next performed to assess the risk factors of GDM. In the discovery phase, circRNAs having fold changes ≥ 2.0 and p-values (t-test) ≤ 0.05 were selected as the significantly differentially expressed ones. In the training and test phase, the Kolmogorov–Smirnov test was used to analyze the normal distribution of the circRNA expression levels, and the paired t test or wilcoxon test was applied when appropriate to evaluate differences in circRNA expression between pregnant women with GDM and non-GDM. CircRNA discrimination potential was analyzed by computing ROC curves and calculating AUCs with corresponding 95% CIs, as well as the optimal specificity and sensitivity values. The relationships between significantly differential expressed circRNA levels and clinical factors of pregnant women with GDM were further analyzed using linear correlation or rank correlation.

## Results

### Sociodemographic factors

Table 2 shows the characteristics and frequency of outcomes of the study. The study cohort comprised a total of 842 subjects, 106 pregnant women terminated their pregnancy during follow-up or withdrew from the cohort due to other conditions, and 736 were available for analyses in this study, among them 106 (14.4%) were diagnosed with GDM. Compared to control subjects, women with GDM showed significantly higher mean age (33.3 ± 4.4 vs. and 31.2 ± 4.2 years), pre-pregnancy BMI (21.7 ± 2.8 vs. 20.5 ± 2.6 kg/m^2^), multipara (68.9% vs. 54.6%) and caesarean section (40.6% vs. 27.8%) (Ps < 0.05). There were no significant differences in other sociodemographic factors and pregnancy outcomes between the GDM and control groups.

**Table 2:** General characteristics of the mothers and newborns in gestational diabetes mellitus (GDM) group and control group.

Variables	GDM (N = 106, %)	Control (N = 630, %)	*P*
**Maternal age (years)**			
$\overline{X}$ ± SD	33.3 ± 4.4	31.2 ± 4.2	< 0.01
**Maternal age group (years)**			
< 35	65 (61.3)	494 (78.4)	< 0.01
≥ 35	41 (38.7)	136 (21.6)	
**Gravidity (times)**			
1	27 (25.5)	201 (32.0)	0.19
≥ 2	79 (74.5)	429 (68.0)	
**Parity (times)**			
0	33 (31.1)	286 (45.4)	< 0.05
≥ 1	73 (68.9)	344 (54.6)	
**Pre-pregnancy BMI (kg/m**^**2**^**)**			
$\overline{X}$ ± SD	21.7 ± 2.8	20.5 ± 2.6	< 0.01
**Gestational weeks (weeks)**			
≥ 37	99 (93.4)	614 (97.4)	0.05
< 37	7 (6.6)	16 (2.6)	
**Delivery mode**			
Vaginal delivery	58 (54.7)	435 (69.0)	< 0.05
Cesarean delivery	43 (40.6)	175 (27.8)	
Other	5 (4.7)	20 (3.2)	
**Neonatal weight (g)**			
$\overline{X}$ ± SD	3260.6 ± 378.4	3229.8 ± 373.9	0.43
**Neonatal sex**			
Male	61 (57.5)	321 (51.0)	0.20
Female	45 (42.5)	309 (49.0)	
**1-min neonatal Apgar scores < 10**			
Yes	92 (86.8)	575 (91.2)	0.14
No	14 (13.2)	55 (8.8)	
**5-min neonatal Apgar scores < 10**			
Yes	80 (75.5)	501 (79.5)	0.34
No	26 (24.5)	129 (20.5)	

In the discovery phase, the study comprised a total of 6 paired of participants (discovery set including 6 GDM subjects and controls, respectively) with lengths of gestation ranging from 15 to 24 weeks, and in the validation phase, there are 12 paired of participants coming from 15 to 24 weeks (training set including 12 GDM subjects and controls, respectively); and there are 18 paired of participants in a test set (Table 1). There were no significant differences in sociodemographic factors and adverse pregnancy outcomes of mother and newborns between the GDM and control groups (Table S2).

### Multivariate Logistic regression of factors associated with GDM

Using a sample size of 736, we performed a multivariable logistic regression analysis to assess the association of GDM with sociodemographic characteristics, clinical variables and biological chemistry parameters, which had previously shown an association with GDM at levels of *P* ≤ 0.05 in the univariate analysis. The multivariate logistic regression models with GDM as the dependent variable demonstrated that maternal age (adjusted relative risk (RR), 2.08; 95% confidence interval (CI) 1.31–3.31) and pre-pregnancy BMI (RR: 1.13, 95% CI 1.05–1.23) were independently significantly associated with high risk of GDM after adjustment for paternal age and maternal factors (Table S3).

### CircRNA expression profiling of pregnant women with GDM and non-GDM

**Figure 1:**
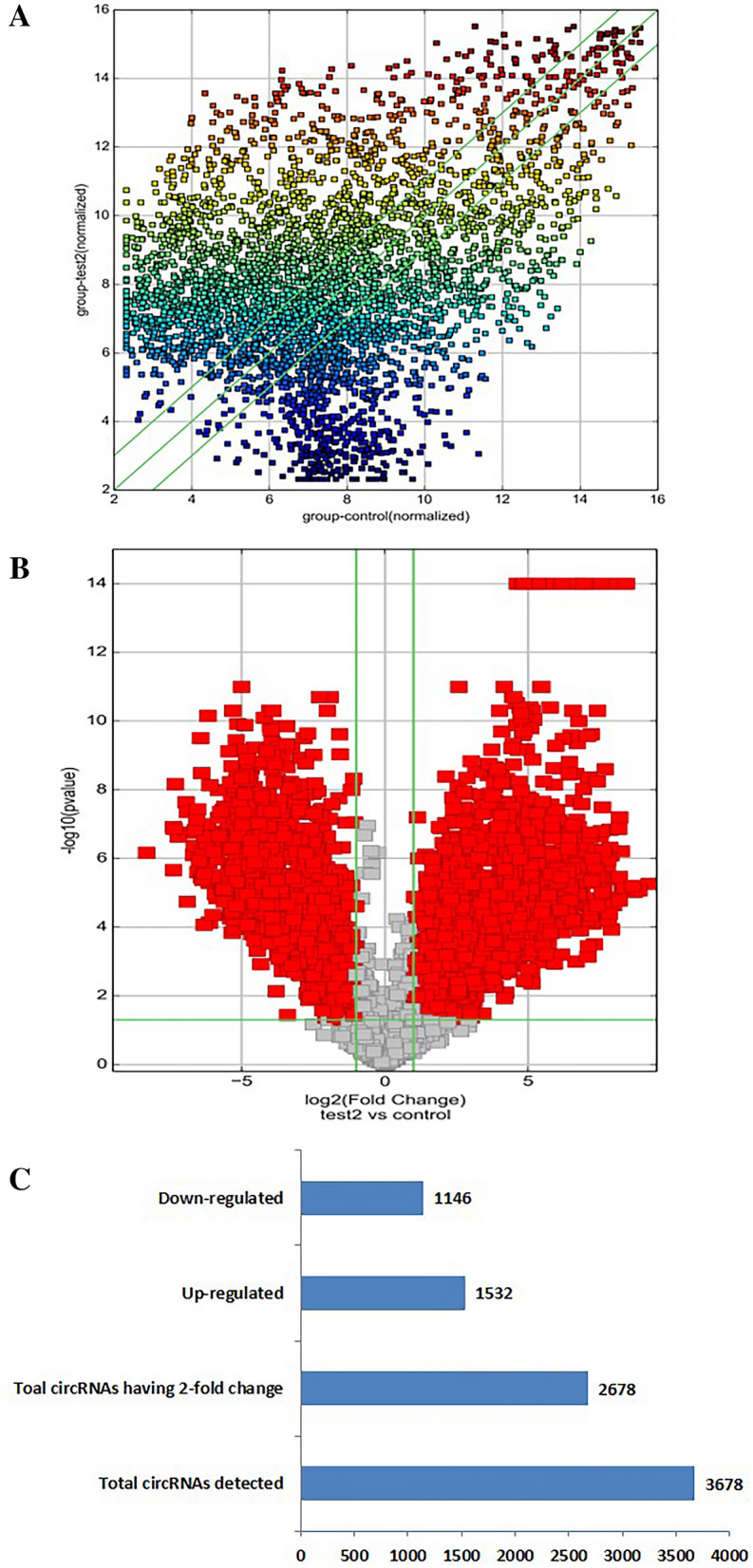
The expression profiles of circRNAs compared between GDM and paired adjacent control group. The Scatter-Plot (**A**) is a visualization method used for assessing the circRNAs expression variation (or reproducibility) between pregnant women with GDM and control group. The X and Y axis values in the scatter-Plot are the normalized signal values of the average normalized signal values (log2 scaling) of each group of samples. The green lines are Fold Change Lines. The circRNAs above the top green line and below the bottom green line indicate that the change of circRNAs in the two compared samples exceeds 2.0 fold. Volcano Plots (**B**): the vertical lines correspond to 2.0-fold up and down, respectively, and the horizontal line represents a p-value of 0.05 (t-test). The red point in the plot represents the differentially expressed circRNAs with statistical significance. Histogram (**C**): among 3678 circRNAs, a total of 2678 circRNAs were detected with 2.0-fold change and p ≤ 0.05 (t-test), of which 1532 were up-regulated and 1146 were down-regulated.

To identify circRNAs associated with GDM, we explored the human circRNAs in 6 pregnant women with GDM and 6 healthy pregnant women from the discovery set with microarrays. Total RNA of the samples was extracted. ArrayStar Human circRNA Array analysis was adopted for profiling the human circRNAs expression. In total, 3,678 circRNAs were detected. After filtering the data to remove circRNAs with low expression, there were 2678 circRNAs exhibiting fold changes ≥ 2.0 and showing significant differential expression (FDR 0.05). The dendrogram of hierarchical clustering analysis shows the relationships among the circRNAs expression patterns between samples, and indicated that circRNA expression profiling separated pregnant women with GDM from health pregnant women (Fig. 1A,B).

To detect significantly deregulated circRNAs in GDM, supervised analysis was performed. A total of 1532 circRNAs were up regulated in pregnant women with GDM, whereas 1146 were down regulated having a twofold to fold-change (FC) in expression levels (Fig. 1C).

The Gene Ontology (GO) analysis provides a controlled vocabulary to describe the differentially expressed transcript attributes in all organisms. We found that the highest enriched GOs targeted by up-regulated transcripts were cellular process (ontology: biological process), cell part (ontology: cellular component) and binding (ontology: molecular function) and that the highest enriched GOs targeted by the down-regulated transcripts were signaling (ontology: biological process), intracellular (ontology: cellular component) and binding (ontology: molecular function) (Fig. S1A,B). Pathway analysis indicated that 53 pathways corresponded to up-regulated transcripts and that the top 10 most enriched network included Calcium signaling pathway—Homo sapiens (human), Rap1 signaling pathway—Homo sapiens (human), Insulin signaling pathway—Homo sapiens (human) , etc. Furthermore, this analysis showed that 22 pathways corresponded to down-regulated transcripts and that the top 10 most enriched network also included Calcium signaling pathway—Homo sapiens (human) (Fig. S1C).

### CircRNA abundance validation by qPCR analysis

CircRNAs with the smallest FDR and highest fold change were selected when comparing pregnant women with GDM and healthy pregnant women and tried to validate them by qPCR. According to the differential expression analysis, 5 of the most differentially abundant circRNAs were selected to be validated in 12 paired of case and control samples (training set) using qPCR. Hence hsa_circRNA_100380 (DCAF6), hsa_circRNA_101232 (ZDHHC20), hsa_circRNA_102893 (CLK1) and hsa_circRNA_103740 (SCLT1) were confirmed to be down regulated in GDM, whereas hsa_circRNA_102736 (XPO1) was up regulated (Fig. S2). Statistical analysis validated that only hsa_circRNA_102893 (CLK1) was confirmed to be significantly down regulated in GDM.

**Figure 2:**
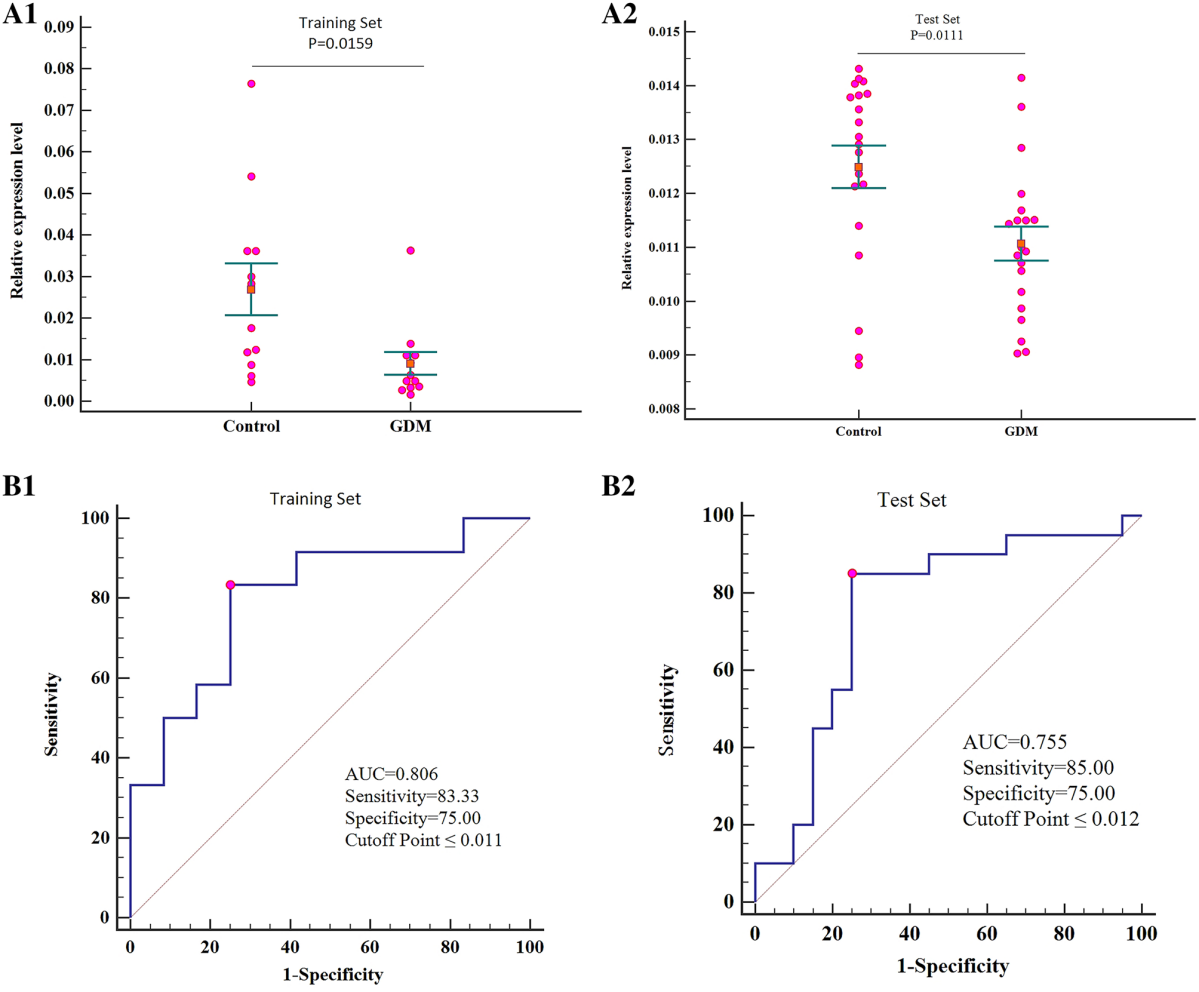
Circulating hsa_circRNA_102893 expressed in the plasma of pregnant women with GDM in comparison with healthy pregnant women (control). (**A**) Relative expression levels of hsa_circRNA_102893 with their corresponding *P* values (Paired t-test) in the training set and test set. Scatter plots show relative expression levels. The orange boxes and the horizontal bars represent the mean and the standard error, respectively. (**B**) ROC curves of hsa_circRNA_102893 and the AUC, sensitivity, specificity and cutoff point in the training set and test set. Red point is corresponding to Youden index.

The significant circRNA identified (hsa_circRNA_102893) was then subjected to confirm using qPCR in another two sets of plasma from 18 paired participants (test set). The results showed a strong consistency among the two data sets. The expression level of hsa_circRNA_102893 was confirmed to be significantly down expressed in the plasma of GDM pregnant women (Fig. 2A1,A2).

### Hsa_circRNA_102893-targeted miRNA-mRNA network prediction and annotation

According to the initial array data analysis, the genomic locus of hsa_circRNA_102893 is on chromosome 2 and the predicted sequence of the best linear transcript is uc002uwe.2. Supposing that hsa_circRNA_102893 is an upstream molecular sponge in its circRNA-miRNA-mRNA network, we predicted a hsa_circRNA_102893- miRNA-gene network using the CircNet database of endogenous RNA functions (https://circnet.mbc.nctu.edu.tw/). Hsa_circRNA_102893 (CLK1) affects the expression of 24 target genes by binding to miR-33a-5p, miR-2115-3p, miR-197-3p, miR-5187-5p and miR-198 (Fig. S3A). CircRNA binding protein prediction was performed using the CircInteractome database (https://circinteractome.nia.nih.gov/), hsa_circRNA_102893 was predicted to have six flanking region binding proteins, including the EIF4A3 protein involved in the translational promoter and the AGO protein involved in the processing and maturation of small RNA (Fig. S3B).

### Potential prediction values of hsa_circRNA_102893 for GDM

ROC curve analysis was performed to evaluate the diagnostic utility of hsa_circRNA_102893 (CLK1). The resulting curves showed that hsa_circRNA_102893 was the valuable biomarker for discriminating GDM from healthy individuals, with AUCs of 0.806 (95% CI 0.594–0.937, P = 0.0011) in validation set, 0.741 (95% CI 0.568–0.872, P = 0.0070) in training set and 0.944 (95% CI 0.652–1.000, P < 0.0002) in test set, and diagnostic sensitivity and specificity at the optimal cutoff of 83.33% and 75.00% for validation set, 83.33% and 72.22% for training set and 83.33% and 100.00% for test set (Table 3, Fig. 2B1,B2). The results of correlation analysis showed that there were no significant differences in hsa_circRNA_102893 expression levels and clinical factors and pregnancy outcomes of pregnant women with GDM (Table S4).

**Table 3:** Diagnostic performance of hsa_circRNA_102893 for the prediction of pregnant women with GDM and healthy pregnant women (Control).

Dataset	Group	n	Se (%)	Sp (%)	+ LR	− LR	AUC (95%CI)	*P*	Criterion value
Training	GDM	12	83.33	75.00	3.33	0.22	0.806 (0.594–0.937)	0.0011	≤ 0.011
	Control	12							
Test	GDM	18	85.00	75.00	3.40	0.20	0.755 (0.593–0.877)	0.0022	≤ 0.012
	Control	18							

*Se* sensitivity, *Sp* specificity, + *LR* positive likelihood ratio, − *LR* negative likelihood ratio.

## Discussion

In this study, we report that hsa_circRNA_102893 may be a potential novel and stable biomarker for early GDM detection. First, we performed unsupervised hierarchical clustering and observed a clear separation between GDM and healthy pregnant women, suggesting that circRNA expression profiling could be used to classify GDM. Second, a supervised analysis was performed to identify circRNAs associated with GDM. The large amount of deregulated circRNAs identified in our sampls of GDM highlights the important role that circRNAs play in GDM development. Third, among the 5 most deregulated circRNAs, hsa_circRNA_102893 (CLK1) was confirmed repeatedly to be significantly down regulated in GDM using qPCR in training set and test set, respectively. Fourth, ROC curve analysis showed that hsa_circRNA_102893 was the valuable biomarker for discriminating GDM from healthy individuals. Hsa_circRNA_102893 hasn’t been reported to be associated with GDM before, and our results expand the knowledge on circRNA deregulation in GDM.

Recently, a large amount of circRNAs in the eukaryotic tree of life have been identified, and several potential functions of circRNAs have been predicted and demonstrated to be miRNA sponges^7,9,19^. Cdr1as was identified to be expressed in islet cells, and its overexpression may inhibit miR-7 function and significantly increased insulin mRNA level and granule secretion in β cells; and Cdr1as/miR-7 is involved in the cAMP and PKC signal pathway^20^.

Initial characterization of circular RNAs and their underlying molecular mechanisms in GDM need to be extensively investigated. In this study, among five selected circRNAs with most differentially abundance, only hsa_circRNA_102893 was confirmed by qPCR to be significantly down-regulated in pregnant women with GDM in both validation set and test set. This was also illustrated by the high efficiency of prediction of GDM by ROC in the two data sets. Age and pre-pregnancy BMI of pregnant women had no impact on the hsa_circRNA_102893 level, which indicates that hsa_circRNA_102893 level is independent of age and pre-pregnancy BMI. This implies that the hsa_circRNA_102893 level can be referenced to other pregnant women without age and pre-pregnancy BMI adjustment. By contrast, age and pre-pregnancy BMI have a strong influence on GDM, which has to be taken into consideration when evaluating disease severity.

Bioinformatics analyses found that hsa_circRNA_102893 harbored binding sites for miR-33a-5p and miR-197-3p. The up-regulation of miR-33 and miR-197 was the result of the down expression of hsa_circRNA_102893. MiR-33a is located in the intron 16 of SREBP-2 which has been proved to preferentially enhance cholesterol synthesis^21^. Circulating miR-33a level is reported to be positively correlated with serum total cholesterol (r = 0.364, p = 0.048) and low-density lipoprotein cholesterol (r = 0.383, p = 0.037) in T2D patients at high risk for developing atherosclerotic cardiovascular diseases^22^. MiR-33 can target silencing of adenosine monophosphate (APM)-activated protein kinase (AMPK) expression, which is the core of research on diabetes^23^.

MiR-197-3p has been reported to have an effect on beta cell function in type 1 and type II diabetes. In a cohort including children with new-onset type 1 diabetes mellitus, the increase of hsa-miR-197-3p levels at 3 months in patients who completed a 5-year follow-up, was associated with a sixfold increase in remaining β-cell function 12 months after diagnosis^24^. In type II diabetes characterized by impaired cell function and insulin resistance, hsa-miR-197-3p was also found to be associated with impaired glycaemic control^25^.

We also found that hsa_circRNA_102893 was predicted to be able to have a binding with EIF4A3. Eukaryotic initiation factor 4A3 (EIF4A3) is an ATP-dependent RNA helicase, which is a core component of exon junction complex (EJC). EJC plays a variety of roles in RNA metabolism, such as RNA translation, monitoring and localization, etc. EIF4A3 has been confirmed to play an important role in post-transcriptional regulation processes^26^. Studies showed that EIF4A3 could be used as a diagnostic marker for various cancers, such as breast cancer^27^, glioblastoma multiforme^28^ and epithelial ovarian cancer^29^. In the occurrence of cancer, EIF4A3 regulates cell cycle and apoptosis via the TNF-α/NF-ĸB signaling pathway and other important signaling pathways, promotes tumor cell migration and invasion, and the formation of drug resistance^30^. Tumor necrosis factor-alpha (TNF-alpha) is an effective mediator of inflammation, inducing the expression of NF-kappaB-mediated gene network. The TNF-α/NF-κB signaling pathway is a key signaling pathway that affects metabolic diseases, including diabetes^31^. EIF4A3 may play an important role in the pathogenesis of GDM. However, little is known about the exact role and underlying mechanism of EIF4A3 in the development and progression of GDM.

The results of qPCR showed that hsa_circRNA_102893 was confirmed to be significantly deregulated in the validation set and test set in GDM. And ROC curve analysis showed the discrimination potential of hsa_circRNA_102893, with AUCs ranging from 0.741 to 0.806 in both the two data sets, supporting the potential valuable biomarker for discriminating pregnant women with GDM from healthy individuals. Therefore, we hypothesized that hsa_circRNA_102893 might be candidates for noninvasive GDM detection.

In conclusion, we have first identified a large number of deregulated circRNAs in GDM, and have reported and validated the down expression of hsa_circRNA_102893 in the plasma of pregnant women with GDM and demonstrated the potential utility of hsa_circRNA_102893 as noninvasive GDM biomarkers. An advantage of this study is that the participants had early-stage gestational period (in the second trimester) at the time of sample collection, which highlights the relevance of the identified circRNAs in early GDM detection. Although these results are promising, prospective studies on larger cohorts of participants are required to confirm the early detection role of hsa_circRNA_102893.

## Supplementary information

Supplementary Information
