# Supplementary material for: Circulating expression of Hsa_circRNA_102893 contributes to early gestational diabetes mellitus detection
Source: Sci Rep. 2020 Nov 4;10:19046. doi: 10.1038/s41598-020-76013-5 (PMC7642424; doi:10.1038/s41598-020-76013-5)
Supplement: Supplementary file 1 — Supplementary Information [file 41598_2020_76013_MOESM1_ESM.pdf]

Circulating Expression of Hsa_circRNA_102893 Contributes to Early Gestational Diabetes Mellitus Detection

Running Title: Hsa_circRNA_102893 Prediction of GDM

Hongling Yang[1,†], Weitao Ye[2,†], Ruihua Chen[3,†], Fangling Zeng[1,†],Yan Long[1], Xiaoyan Zhang[1], Jun Ma[2], Qiangsheng Gan[2], Rehemayi·rehemutula[2], Chunyan Zhu[2,*]

[1] Department of Laboratory, Guangzhou Women and Children's Medical Centre, Guangzhou Medical University, Guangzhou, China

[2] Department of preventive medicine, School of Public Health, Guangzhou Medical University, Guangzhou, 511436, China

[3] Department of health education, Health Education Center of Panyu, Guangzhou, China

* **Correspondence to**: Chunyan Zhu

Xinzao, Panyu District, Guangzhou, 511436, P.R. China

Tel: +86-20-37103520, Fax: +86-20-37103520

E-mail: zchyan@163.com

† The author should be regarded as joint First Authors.

Table S1 Sequence of the internal reference and the paired primers of qPCR

circRNA	Gene symbol	Bidirectional primer sequence	Annealing temperature (℃)	Product length (bp)
β-actin（H）		F:5' GTGGCCGAGGACTTTGATTG 3'<br>R :5’ CCTGTAACAACGCATCTCATATT 3’	60	73
hsa_circRNA_103740	SCLT1	F:5'ACAACTCCGAAAAAAACTTAGGTTG 3'<br>R :5’ CTGTGCCCAGGGGAAAGG 3’	60	83
hsa_circRNA_102893	CLK1	F:5' GATGACGAACATCACAGTACAT 3'<br>R:5’TCTGTGTGAGTCAACTTATTACTGT 3’	60	95
hsa_circRNA_101232	ZDHHC20	F:5' ACCTATCTATACCACATCAGCT 3'<br>R :5’ CAAACATAACAAAGAACAGATG 3’	60	107
hsa_circRNA_100380	DCAF6	F:5' ACAGATTGTATCCTGCTCTG 3'<br>R :5’ TTAATCTTTGGATAAATTCTCTT 3’	60	139
hsa_circRNA_102736	XPO1	F:5' ATACGAAATACTATGGACTACAA 3'<br>R :5’ TCTTGAGCCATTCTTTGC 3’	60	98

Table S2 Sociodemographic characteristics of the mothers and newborns with gestational diabetes mellitus (GDM) or not (controls) in the discovery set, training set and test set.

		Discover y set		Taining set		Test set	
		GDM (n = 6)	Control (n = 6)	GDM (n = 12)	Control (n = 12)	GDM (n = 18)	Control (n = 18)
Maternal age (years) #	Mean± SD	31.3±3.1	32.2±4.1	31.8± 3.3	32.3± 3.5	29.6±3.1	29.6±3.3
	Min-max	26-35	27-38	26-38	27-38	24-38	24-38
Gravidity (times) #	1	2(33.3)	2(33.3)	3(25.0)	4(33.3)	17(53.1)	17(53.1)
	≥ 2	4(66.7)	4(66.7)	9(75.0)	8(66.7)	15(46.9)	15(46.9)
Parity (times) #	0	4(66.7)	6(100.0)	5(41.7)	5(41.7)	23(71.9)	25(78.1)
	≥ 1	2(33.3)	0	7(58.3)	7(58.3)	9(28.1)	7(21.9)
Pre-pregnancy BMI (weight/height^2)	< 24	5(83.3)	5(83.3)	11(91.7)	8(66.7)	30(93.7)	27(84.4)
	≥ 24	1(16.7)	1(16.7)	1(8.3)	4(33.3)	2(6.3)	5(15.6)
Cesarean delivery	No	2(33.3)	4(66.7)	6(50.0)	6(50.0)	20(62.5)	26(81.3)
	Yes	4(66.7)	2(33.3)	6(50.0)	6(50.0)	12(37.5)	6(18.7)
Newborn birth weight(g)	Mean± SD	3086±249	3215±245	3032.5±642.8	3212.5± 473.7	3177.8±492.1	3210.9±388.6
Newborn sex	Male	2(33.3)	5(83.3)	7(58.3)	5(41.7)	17(53.1)	15(46.9)
	female	4(66.7)	1(16.7)	5(41.7)	7(58.3)	15(46.9)	17(53.1)
Intensive neonatal care	No	6(100)	6(100)	12(100)	12(100)	31(96.9)	32(100)
	Yes	0	0	0	0	1(3.1)	0
Premature delivery (<37 weeks)	No	0	0	11(91.4)	12(100)	29(90.6)	32(100)
	Yes	6(100)	6(100)	1(8.3)	0	3(9.4)	0
BS0	Mean± SD	4.5±0.5	4.4±0.4	4.4±0.5	4.4±0.4	4.6±0.5*	4.3±0.4
BS60	Mean± SD	9.6±2.0*	7.1±1.4	9.1±1.8**	7.1±1.3	9.4±1.9**	7.2±1.3
BS120	Mean± SD	8.6±1.3**	6.3±1.1	8.8±1.4**	6.5±0.9	8.5±1.6**	6.3±0.9

# Maternal age, gravidity, parity and gestational weeks were determined at the time of recruitment. Control subjects were matched against subjects with GDM.

* P<0.05, **P<0.01

Table S3 Association between maternal sociodemographic characteristics, clinical parameters and GDM of multivariate logistic regression analysis

Variable	β	S.E.	*P*	RR*	95% CI (RR)
Maternal age (years)	0.73	0.24	<0.01	2.08	1.31-3.31
Prepregnancy BMI (weight/height^2)	0.13	0.04	<0.01	1.13	1.05-1.23
APTT*	-0.08	0.06	0.14	0.92	0.82-1.03
FIB*	-0.02	0.19	0.91	0.98	0.67-1.43
MONO*	1.84	0.96	0.05	6.31	0.97-41.18
NEUT*	0.09	0.08	0.23	1.09	0.94-1.27
HCT*	0.08	0.06	0.14	1.09	0.97-1.22
Constant	-5.38	0.86	<0.01	0.01	

* Data for first pregnancy or second trimester

APTT: Activated Partial Thromboplastin Time, FIB: Fibrinogen, MONO: Monocyte, NEUT: Neutrophils, HCT: Hematocrit, RR: relative risk.

Table S4 The relationship of hsa_circRNA_102893 expression levels in plasma with clinical factors and pregnancy outcomes of pregnant women with GDM

Variables	GDM		Control	
	Correlation Coefficient	*P*	Correlation Coefficient	*P*
Maternal age (years)	0.426	0.1674	-0.592	0.0425
Prepregnancy BMI (weight/height^2)	0.025	0.8951	0.269	0.3966
Gestational weeks (weeks)	0.068	0.7100	-0.243	0.1811
Neonatal weight (g)	0.109	0.5513	-0.259	0.1526
BS0	0.548	0.0650	0.136	0.5353
BS60	0.203	0.2821	-0.223	0.3061
BS120	0.054	0.7768	-0.248	0.2539

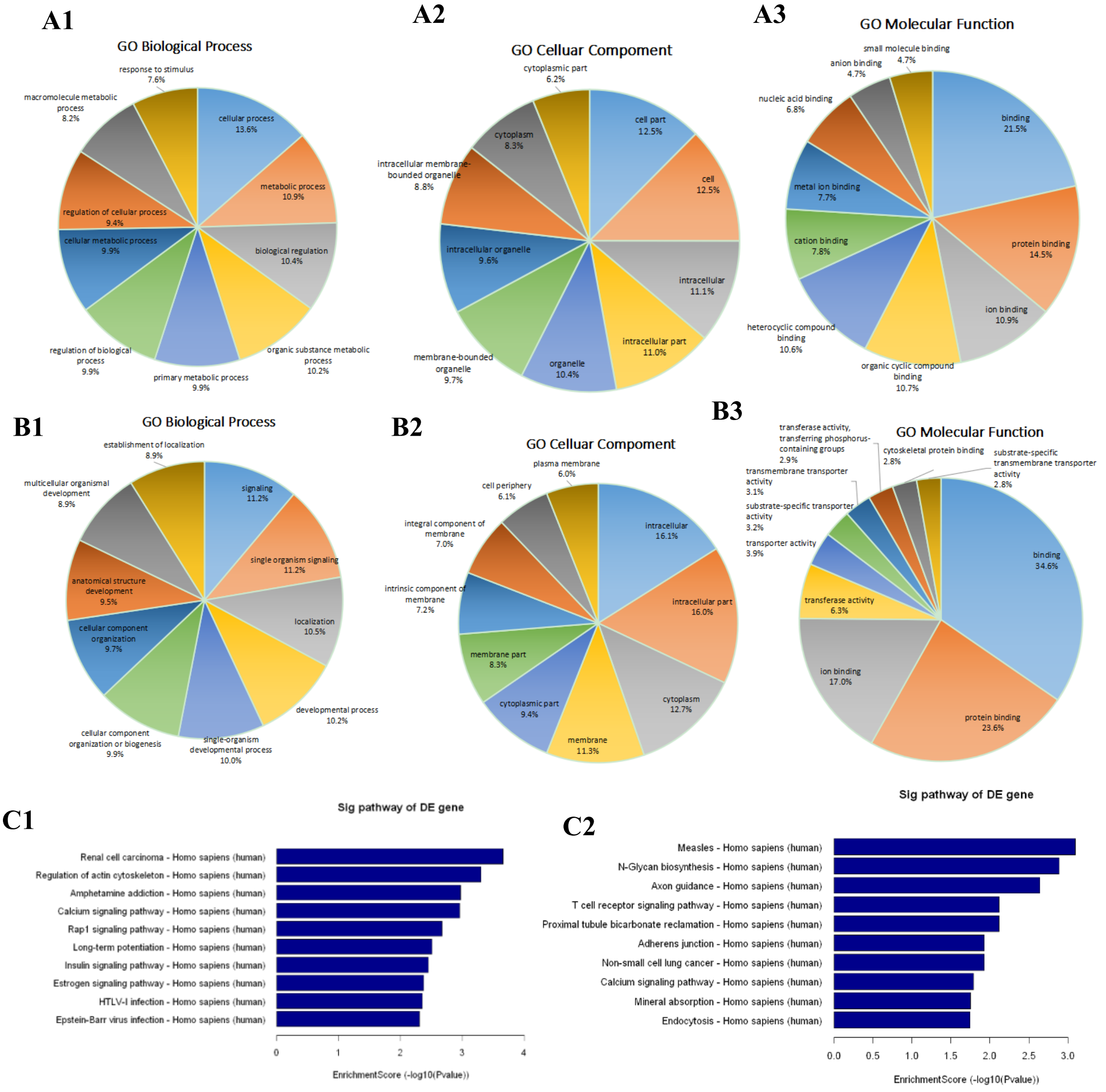

Fig S1. The bioinformatic analysis of the differentially expressed profiles. The pie charts show the top ten counts of the significant enrichment terms in differentially expressed up-regulated circRNAs (A1-A3) and down-regulated circRNAs (B1-B3). The bar plots show the top ten Enrichment score (-log10 (P-value)) value of the significant enrichment pathway in differentially expressed up-regulated circRNAs (C1) and down-regulated circRNAs (C2).

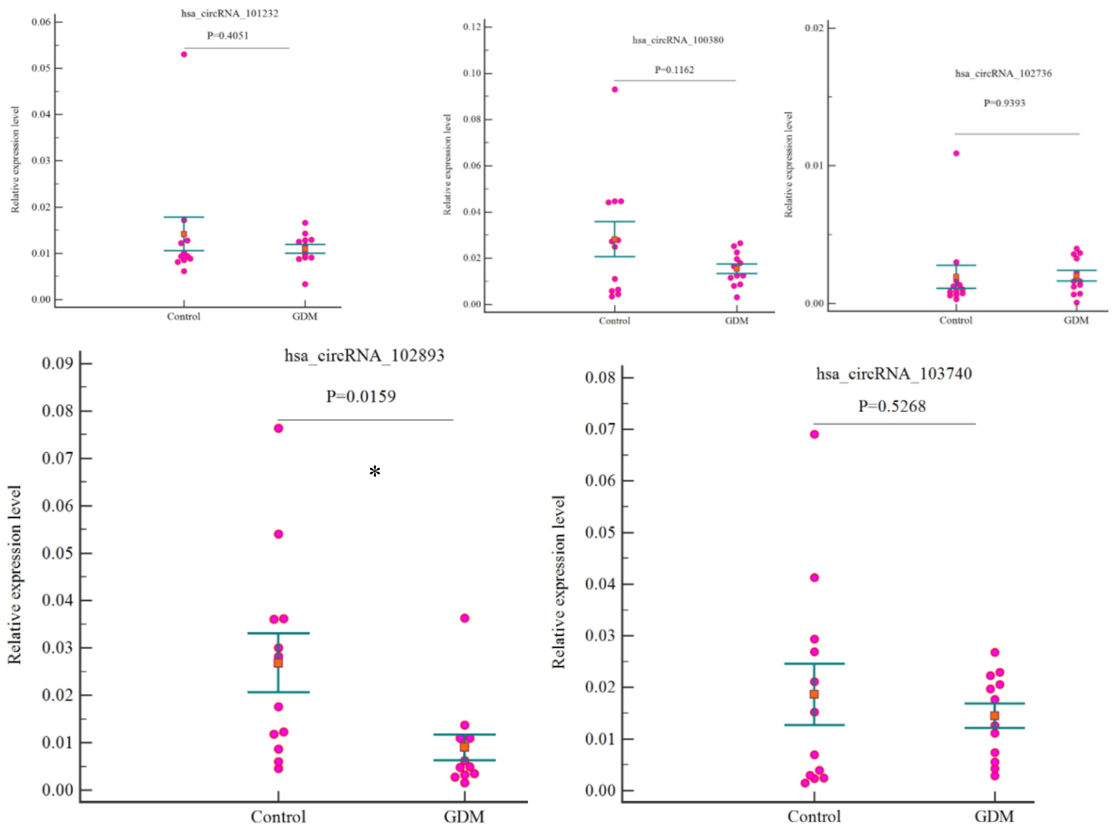

Fig S2. Validation of most relevant circRNAs by qRT-PCR in the plasma of pregnant women with GDM in comparison with healthy pregnant women (control). Scatter plots show relative expression levels of circulating hsa_circRNA_100380, hsa_circRNA_101232, hsa_circRNA_102736, hsa_circRNA_102893 and hsa_circRNA_103740 with their corresponding *P* values (Paired t-test), the orange boxes and the horizontal bars represent the mean and the standard error, respectively. '*'denotes $P<0.05$.

A

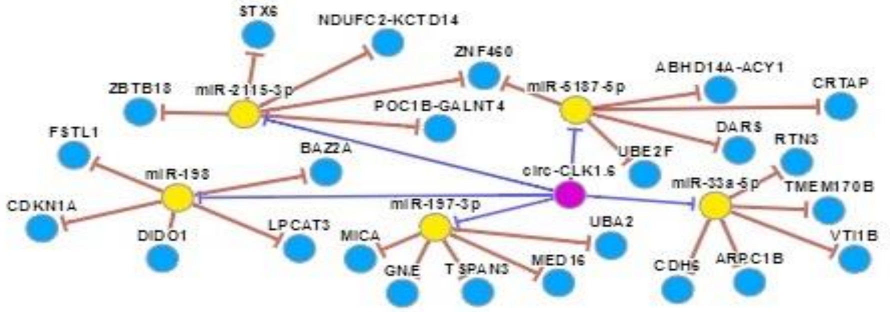

B

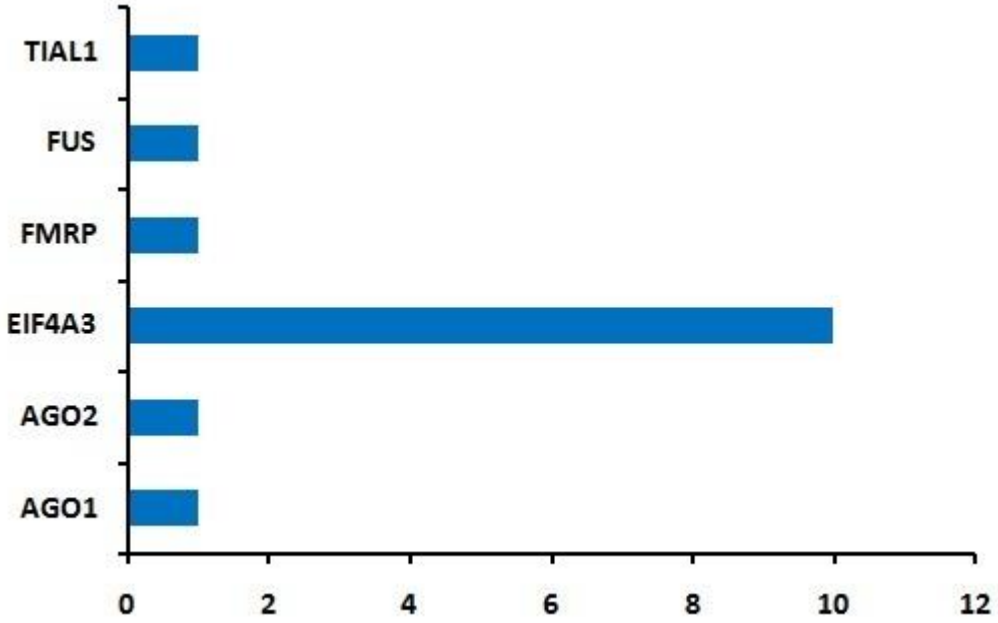

Fig S3. Hsa_circRNA_102893-targeted miRNA-mRNA network prediction and annotation. (A) The miRNA-mRNA network of hsa_circRNA_102893 (CLK1). (B) RNA-binding proteins and sites in the circRNA flanking regions of hsa_circRNA_102893.
